# Hollow Core Bragg Fiber-Based Sensor for Simultaneous Measurement of Curvature and Temperature

**DOI:** 10.3390/s21237956

**Published:** 2021-11-29

**Authors:** Zongru Yang, Weihao Yuan, Changyuan Yu

**Affiliations:** Photonics Research Centre, Department of Electronic and Information Engineering, The Hong Kong Polytechnic University, Hong Kong, China; zongzong.yang@connect.polyu.hk (Z.Y.); weihao.yuan@connect.polyu.hk (W.Y.)

**Keywords:** hollow core Bragg fiber (HCBF), anti-resonant reflecting optical waveguide (ARROW), curvature, temperature

## Abstract

In this paper, the hollow core Bragg fiber (HCBF)-based sensor based on anti-resonant reflecting optical waveguide (ARROW) model is proposed and experimentally demonstrated for simultaneous measurement of curvature and temperature by simply sandwiching a segment of HCBF within two single-mode fibers (SMFs). The special construction of a four-bilayer Bragg structure provides a well-defined periodic interference envelope in the transmission spectrum for sensing external perturbations. Owing to different sensitivities of interference dips, the proposed HCBF-based sensor is capable of dual-parameter detection by monitoring the wavelength shift. The highest curvature sensitivity of the proposed sensor is measured to be 74.4 pm/m^−1^ in the range of 1.1859–2.9047 m^−1^ with the adjusted R square value of 0.9804. In the meanwhile, the best sensitivity of temperature sensing was detected to be 16.8 pm/°C with the linearity of 0.997 with temperature range varying from 25 to 55 °C. Furthermore, with the aid of the 2 × 2 matrix, the dual demodulation of curvature and temperature can be carried out to realize the simultaneous measurement of these two parameters. Besides dual-parameter sensing based on wavelength shift, the proposed sensor can also measure temperature-insensitive curvature by demodulating the intensity of resonant dips.

## 1. Introduction

Optical fiber-based sensors have aroused significant interest in the past few decades, owing to their superiority in the measurement of physical parameters with unique advantages of easy fabrication, compact configuration, inexpensiveness, high sensitivity, and electromagnetic immunity. The simultaneous measurement of curvature and temperature of fiber-optic sensors is an essential application in various fields, such as robotic arms [1], civil engineering infrastructural health monitoring [2], and aerospace composite structures [3]. Furthermore, optical fiber curvature sensors have been developed as wearable devices for movement monitoring [4] and shape sensors for medical instruments [5], which demonstrates their strong practicality and innovation for curvature sensing applications. In recent years, researchers have investigated a variety of optical fiber structures for curvature sensing. Dong et al. (2018) proposed a curvature fiber sensor based on the cascaded interferometers and the curvature sensitivity reached 4.362 nm/m^−1^ within 0–1.134 m^−1^ [6]. Hu et al. (2018) reported a heterostructure cladding solid-core photonic bandgap fiber with a curvature sensing sensitivity of 24.3 nm/m^−1^ in the range of 0–1.75 m^−1^ [7]. Arrizabalaga et al. (2020) used a multi-core fiber to achieve a sensitivity of 17.5 nm/m^−1^ as a curvature sensor [8]. However, the above-mentioned schemes based on multimode interference have not addressed the high cross-sensitivity of temperature. The temperature–curvature cross-sensitivity can severely affect the interference pattern of the output signals and further bring ineluctable errors on curvature measurement. Another scheme based on fiber gratings has also been carried out, including fiber Bragg grating (FBG) [9], long-period grating (LPG) [10], and tilted fiber Bragg grating [11]. Nevertheless, fiber gratings generally require complicated and expensive phase masks in the process of fabrication.

Because photonic sensors are usually susceptible to other undesired perturbations, multi-parametric or temperature-independent detection is of practical significance for curvature sensing. Wu et al. (2017) coupled twin-core fiber and multimode fiber (MMF) to realize the simultaneous detection of curvature and temperature using a coefficient matrix, and the proposed sensor gained a curvature sensitivity of 103.35 nm/m^−1^, ranging from 0.24 m^−1^ to 0.6 m^−1^ [12]. However, the small measurement range limits its application. Gong et al. (2011) generated an all-fiber structure curvature sensor based on MMF with three notches in the transmission spectrum [13], and they stated the possibility for simultaneous monitoring of curvature and other measurands according to weak curvature response of a certain transmission notch. By demodulating different dependences of several transmission notches and curvatures, they obtained discrete curvature sensitivities among the curvature measurement range, which increases the complexity and instability of interrogation. Overall, dual demodulation of curvature and temperature with relatively easy manufacturing as well as large curvature sensing range is vital for fiber-optic curvature sensing with high precision and practicability.

To date, the research has tended to focus on hollow core fibers (HCFs) that guide light in the air core. Two categories of HCFs have been developed, that is, photonic bandgap (PBG) and anti-resonant reflecting optical waveguides (ARROWs) [14], conforming to the light confinement mechanism. Additionally, ARROW-HCFs have been demonstrated as marvelous candidates for optical fiber sensors by virtue of their exclusive properties such as easy fabrication and flexible design [15,16,17]. The hollow core capillary (HCC) is the elementary type of HCFs, which contains a single cladding layer drawn from a pure silica tube. The HCC has exhibited its ability as a curvature sensor [18]; however, a previous work [19] shows that the large transmission loss and low visibility of the HCC spectrum will influence the measurement result.

In this paper, we propose and experimentally demonstrate a fiber sensor based on ARROW-hollow core Bragg fiber (HCBF) for simultaneous measurement of curvature and temperature. The homemade HCBF is comprised of an array of high- and low-index layers surrounding an air core, which offers a clearer spectrum fringe compared to the HCC-based sensor benefiting from the lower transmission loss and smoother envelope of the HCBF at the guided band. A segment of the HCBF was simply sandwiched between two single-mode fibers (SMFs) to construct the inline sensor. Based on the ARROW mechanism, the period lossy dips can be observed in the transmission spectrum of the proposed sensor. Meanwhile, the wavelength and amplitude of the lossy dips show different responses to the variations of curvature and temperature, which means that these two parameters can, in turn, be obtained by monitoring the alteration of the lossy dips. After extracting the dips information, the linear wavelength shift and amplitude change are achieved. Furthermore, the 2 × 2 matrix was used to demodulate the cross-sensitivity between curvature and temperature. The proposed inline dual-parameter HCBF-based sensor possesses the advantages of novelty, low cost, and simplicity.

## 2. Structure, Fabrication and Properties of HCBF

### 2.1. Structure

As a special type of hollow core photonic crystal fibers (HCPCFs), HCBFs can confine the input light in the air core based on the ARROW model. Different from the PBG fiber with complex air-holes cladding, the HCBF is formed by simplified multi-bilayers cladding. The multi-bilayers configuration consists of two media with slightly different refractive index (RI) values, which act as a Bragg reflector to ensure a lower fiber modal loss. Since the transmission loss of the HCBF will reduce with the increasing quantity of bilayers, the HCBF with multi-bilayers cladding is largely used for high-power delivery [20]. However, for optical fiber sensors, we commonly expect that light at specific wavelengths can leak out from the cladding to interact with the perturbations in the external environment. Considering this, the number of bilayers in the cladding should be properly controlled to ensure a relatively high transmittance and strong light–matter interaction in the meantime.

**Figure 1:**
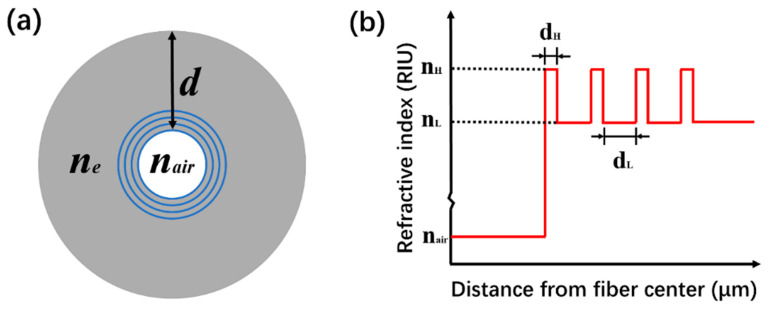
(**a**) Schematic diagram of the cross-section of the HCBF; (**b**) refractive index distribution along the fiber radial direction.

Therefore, in this work, we design four-bilayers cladding HCBF that can weakly confine the light in the air core, which enables the HCBF sensor to present fine fringes of transmission spectrum and relatively low transmission loss. The cross-section view of HCBF is presented in Figure 1a. The fiber is formed by an air core with a diameter of 32 μm, surrounded by four-bilayers annular rings structure with alternant high-low RIs distribution. Blue rings and grey rings represent the high-index and low-index layers, respectively. *n_e_* and *n_air_* are the effective RIs of the cladding, and the air core and *d* is the thickness of the cladding. The RI distribution along the fiber radial direction is depicted in Figure 1b, where a periodic RI profile can be observed. The high-index media are germanium (Ge)-doped silica with *n_H_* = 1.454 at the wavelength of 1550 nm. The low-index media and outer cladding are pure silica with *n_L_* = 1.444 at a wavelength of 1550 nm. Average thicknesses of the high-index layer and low-index layer are $d_{H}=1.06\;\mu m$ and $d_{L}=3.07\;\mu m$, respectively.

### 2.2. Fabrication

The four-bilayer Bragg structure preform was produced using the modified chemical vapor deposition (MCVD) method. Firstly, the pure quartz tube (Heraeus F300, Heraeus Conamic, Shanghai, China) with inner/outer diameter of 25/31 mm was fixed in the CVD equipment. Then, the Ge-doped silica and pure silica were deposited on the inner side of the quartz tube orderly with the thickness of 380 µm and 1000 µm, respectively. The aforementioned processes were repeated 4 times to construct the four-bilayer high-/low-index structure. After that, a preform with a diameter of 13/50 mm can be obtained via the rod-in-tube process. The top of the preform was sealed, and negative pressure was pumped during the drawing process to control the diameter of the hollow core. Finally, the preform was drawn as the HCBF with the core/cladding diameter of 32/125 µm through the modified fiber drawing tower. The detailed information can be found in [21].

**Figure 2:**
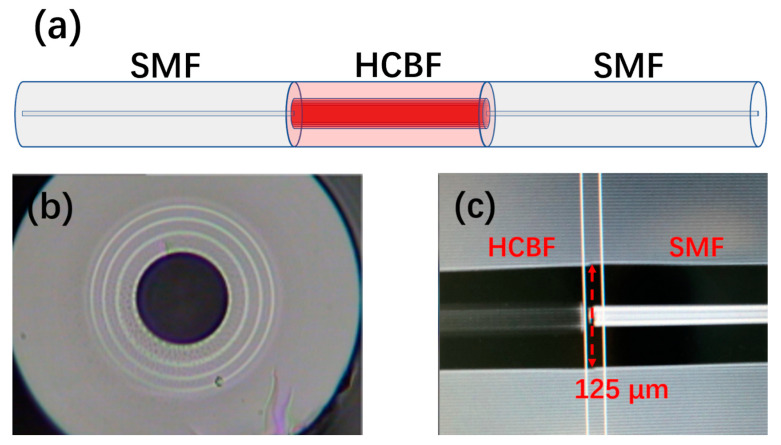
(**a**) Schematic diagram of the SMF–HCBF–SMF structure sensor; (**b**) microscopy image of the cross-section of the HCBF; (**c**) microscopy image of the splicing joint between the HCBF and SMF.

As shown in Figure 2a, the HCBF-based sensor is simply composed of SMF-HCBF-SMF structure without mismatch due to their identical diameters. The commercial fusion splicer (FITEL, S178A, FURUKAWA, Tokyo, Japan) was used to fuse SMF and HCBF. To ensure the ideal mechanical strength of the splicing joint and non-deformation of the air core, the arc power, arc duration, and repetition time were optimized. It is noted that the fiber alignment in the splicing process must be a manual operation, since the splicer cannot identify the hollow core fiber automatically. Figure 2b illustrates the microscopy image of the cross-section of the HCBF where distinct media with different RIs can be clearly observed, and the uniform structure validates the effectivity of the Bragg fiber fabrication. The microscopy image of the splicing joint implies that no air core collapsing occurs between the SMF and HCBF, which is shown in Figure 2c.

### 2.3. Property

**Figure 3:**
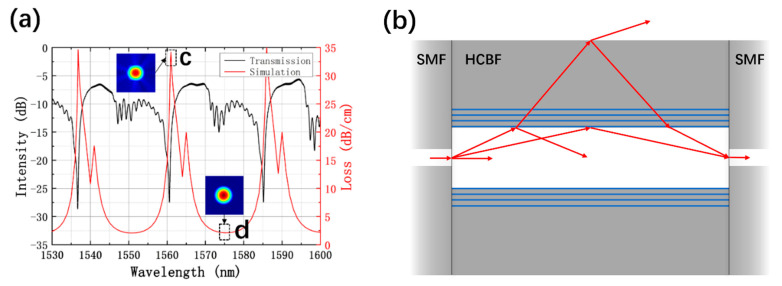
(**a**) Experimental transmission spectrum of the HCBF-based sensor (black curve) and simulation transmission loss spectrum of the HCBF (red curve), insets show the simulated mode profiles at the wavelength of 1561 nm (peak c) and 1575 nm (peak d); (**b**) schematic diagram of the guiding mechanism of the HCBF-based sensor.

Figure 3a shows the experimental transmission spectrum of the HCBF-based sensor with 6 mm HCBF (black curve). The data was collected using a broadband light source (BBS) with a spectral range of 1528–1603 nm and an optical spectrum analyzer (OSA, YOKOGAWA, AQ6374, Tokyo, Japan) with a resolution of 0.05 nm. Three major lossy dips can be distinctly observed at wavelengths of 1536.7 nm, 1560.6 nm, and 1585.1 nm, with an average extinction ratio (ER) as high as 21.51 dB. Additionally, free spectral ranges (FSRs) between adjacent lossy dips are 23.91 nm and 24.56 nm, respectively. Meanwhile, the loss spectrum simulation of the fundamental mode of the HCBF was implemented using the full-vector finite element method (FEM) to analyze its propagation property, as shown in the red curve of Figure 3a. Calculated FSRs are found to be 24.2 nm and 25.0 nm, which matches well with the experiment results. Moreover, it can be seen that the waveband far away from the lossy dip wavelengths exhibits strong light confinement, which can be attributed to the four-bilayer Bragg structure (i.e., 2.14 dB/cm at a wavelength of 1575 nm). Simulated mode profiles at wavelengths of 1561 nm and 1575 nm are plotted as insets in Figure 3a, which correspond to losses at the maxima and the minima, respectively. The fine geometry of Gaussian beam (peak d) at a wavelength of 1575 nm indicates the well light confinement. In contrast, leakage of light (peak c) can be clearly seen at a wavelength of 1561 nm, with a transmission loss of 34.19 dB/cm.

The light-guiding mechanism of the HCBF-based sensor is presented in Figure 3b. The multi-modes are excited at the splicing joint due to the mode mismatch while the light is guided from the SMF to the HCBF. According to the ARROW model, the fiber cladding can be considered a Fabry–Pérot (FP) etalon. A part of the light will be refracted into the cladding, because $n_{air}$ is less than $n_{e}$, and propagation paths may be divided into three groups: (1) the light that meets the resonant condition in fiber cladding will spread out of the HCBF, which is called the resonant wavelength, corresponding to sharp periodic lossy dips shown in Figure 3a; (2) otherwise, the light that does not meet the resonant condition will be reflected as well as confined to the air core and keeps propagating, which is called the anti-resonant wavelength, corresponding to the guided band experiencing low leakage; and (3) part of the guided modes go through multiple reflections on inner/outer interfaces of the cladding, resulting in interference, which takes shape in ripples as revealed in the experimental transmission spectrum. The resonant wavelength of the ARROW-HCBF can be expressed as [22]

(1) $$\lambda_{m}=\frac{2d}{m}\sqrt{n_{e}^{2}-n_{air}^{2}},$$

where m is an integer beginning at 1, d is the thickness of the cladding, and $n_{e}$ and $n_{air}$ are the effective RIs of the cladding and air.

According to Equation (1), the variation of RIs ($n_{e}$ and $n_{air}$) and the thickness (d) of fiber cladding will result in the wavelength shift of resonant dips. Considering the sensor’s response to temperature change, the thermal–optic coefficient and thermal expansion coefficient of the air are negligible compared to that of the silica cladding. Furthermore, the thermo-optic effect of fiber cladding is dominant, while the effect of thermal expansion is ignorable. Therefore, the wavelength shift caused by temperature change is mainly attributed to the variation of effective RI ($n_{e}$) of the cladding. For the relationship between curvature and wavelength shift, the bending-induced deformation of structural symmetry may also perturb the effective RI ($n_{e}$) of the fiber cladding. In general, the curvature and temperature sensitivities of the HCBF may be attributed to the change of $n_{e}$. The temperature- and curvature-induced wavelength shift can be simply derived from Equation (1) as

(2) $$\Delta \lambda_{m}=\frac{2d}{m\sqrt{n_{e}^{2}-n_{0}^{2}}}\cdot \frac{\partial n_{e}}{\partial T}\cdot \Delta T+\frac{2d}{m\sqrt{n_{e}^{2}-n_{0}^{2}}}\cdot \frac{\partial n_{e}}{\partial C}\cdot \Delta C,$$

where ΔT and ΔC are the change of temperature and the change of curvature, respectively. The coefficient $2d/\left(m\sqrt{n_{e}^{2}-n_{0}^{2}}\right)$ can be considered a constant within a certain measurement region. According to Equation (2), the change of resonant wavelengths is linearly proportional to the variation of temperature with a slope coefficient $\partial n_{e}/\partial T$, which is described as the thermo-optic coefficient. Additionally, the relationship between the wavelength shift of resonant dips and curvature depends on the coefficient $\partial n_{e}/\partial C$. Since the strain RI coefficient is negative for silica fiber [23], the resonant dip is supposed to experience a blueshift with curvature increasing. Basically, the different values of m and $n_{e}$ suggest that different sensitivities can be gathered in the course of observing the wavelength shift of different dips under the same condition, which means the 2 × 2 matrix can be used to measure the curvature and temperature simultaneously.

## 3. Curvature and Temperature Sensing Experiment

### 3.1. Curvature Sensing

**Figure 4:**
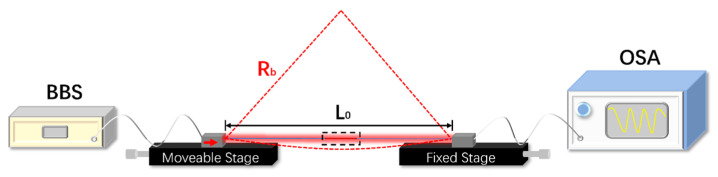
Schematic diagram of the experimental setup of the HCBF-based sensor for curvature measurement.

The schematic diagram of the experimental setup for curvature sensing using the SMF–HCBF–SMF sensor is shown in Figure 4. Both ends of the fiber sensor were secured horizontally by a couple of fiber holders with the original distance of $L_{0}=8\;\mathrm{cm}$ (straight fiber without stretching) to ensure sufficient curvature range, and the HCBF (black dashed box) was set in the middle of two fiber holders. Each fiber holder was fixed on a one-dimensional (1D) translation stage. One of the 1D translation stages was set as the moveable stage with the moving step of 30 µm toward the fiber axial direction (red arrow), while another was set as the fixed stage. The light launching from the BBS was transmitted through the fiber sensor and then collected by the OSA, which is applied to analyze the transmission spectrum. The red dotted curve indicates the fiber sensor after bending, and the curvature can be obtained by the following equation [24]:

(3) $$C=\frac{1}{R_{b}}≅\sqrt{\frac{24x}{L_{0}^{3}}},$$

where C is the curvature, $R_{b}$ is the radius of curvature, and *x* is the moving distance toward the fiber axial.

It is worth noting that the wavelength of resonant dips is irrelevant to the length of the HCBF according to Equation (1). Several experiments were carried out with various lengths of HCBF under the same experimental conditions, and results proved that the wavelength of resonant dips nearly remained unchanged. To avoid excessive transmission loss, the length of HCBF used in the experiment is 6 mm to guarantee a relatively large transmittance and the applicable visibility of the spectrum.

**Figure 5:**
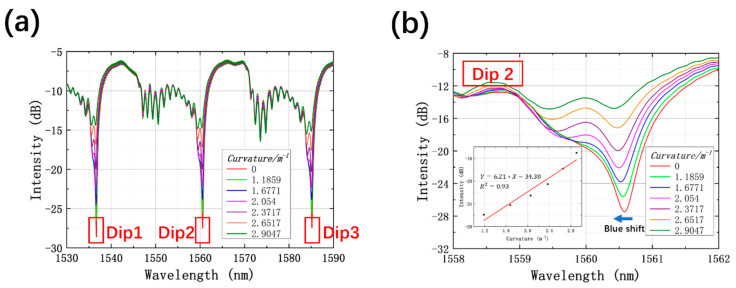
(**a**) Transmission spectra of the HCBF-based sensor under different curvature values; (**b**) enlarged transmission spectra in the region of Dip 2, inset shows the relationship between intensity of Dip 2 and curvature.

The total displacement length of the experiment for curvature sensing was 180 µm with the moving step x of 30 µm, corresponding to curvature values of 0, 1.1859, 1.6771, 2.0540, 2.3717, 2.6517, and 2.9047 m^−1^. Transmission spectra under multiple curvature values are depicted in Figure 5a. Three sharp resonant dips in the range from 1530 to 1590 nm can be detected, which are designated as Dip 1, Dip 2, and Dip 3 around wavelengths of 1536.6, 1560.5, and 1585.1 nm, respectively. From the enlarged interference pattern of Dip 2 shown in Figure 5b, as curvature rising, the lossy dip reveals a steady blueshift accompanied by the marked reduction in loss intensity. As such, other dips show similar phenomena, which is consistent with Equation (2). The inset in Figure 5b depicts the linear relationship between the loss intensity of Dip 2 and curvature ranging from 1.1859 to 2.9047 m^−1^. The fitting slope is 6.21 dB/m^−1^, with a linearity of 0.93. The amplitude change of resonant dips may owe to the destruction of the propagating symmetry of the interference beams. The response of loss intensity to curvature change is remarkable, which is advantageous to curvature sensing.

**Figure 6:**
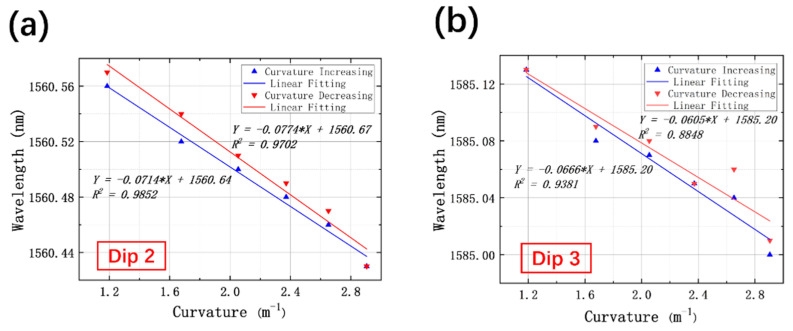
Relationship between the wavelength of resonant dips and curvature with curvature increasing and decreasing for (**a**) Dip 2 and (**b**) Dip 3.

To realize the simultaneous measurement of curvature and temperature, Dip 2 and Dip 3 were selected for analysis. The experiments were repeated with curvature increasing and decreasing within 1.1859–2.9047 m^−1^, and the linear fittings of Dip 2 and Dip 3 are displayed in Figure 6a,b, respectively. For Dip 2, with curvature increasing, the resonant wavelength exhibits a linear blueshift. The sensitivity of Dip 2 in the process of curvature increasing is measured to be −71.4 pm/m^−1^, with adjusted R square values of 0.9852. Inversely, the redshift of resonant wavelength occurs when curvature reduces, and the calculated slope and linearity are −77.4 pm/m^−1^ and 0.9702, respectively. Similarly, the sensitivities of Dip 3 for curvature sensing are measured to be −66.6 pm/m^−1^ as curvature increases and −60.5 pm/m^−1^ as curvature decreases, with adjusted R square values of 0.9381 and 0.8848, respectively. The relatively low linearity of Dip 3 is supposed to be a result of errors introduced in the course of drawing and fabrication processes, which leads to the asymmetry of the air core as well as the nonuniformity of each cladding layer. In addition, bending may bring the structural distortion of the splicing joint between SMF and HCBF due to the slight fusing mismatch during the splicing process. Another assumption is that the incongruity of the SMF–HCBF–SMF structure can probably lead to a disturbance of curvature sensing.

### 3.2. Temperature Sensing

**Figure 7:**
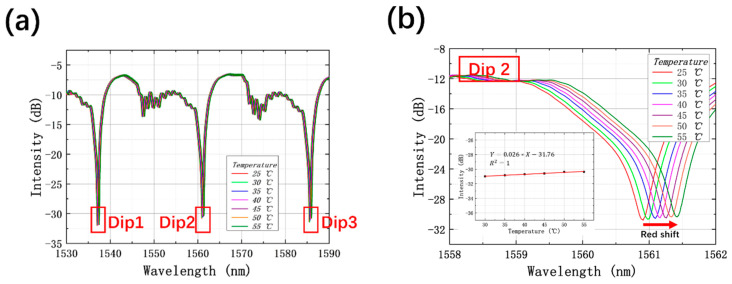
(**a**) Transmission spectra of the HCBF-based sensor under different temperature values; (**b**) enlarged transmission spectra in the region of Dip 2, inset shows the relationship between the intensity of Dip 2 and temperature.

For temperature sensing, the HCBF-based sensor was located in a furnace, and the temperature was set varying from 25 to 55 °C with a step of 5 °C. Figure 7a shows the interference spectra with three resonant dips, denominated as Dip1, Dip2, and Dip 3, which are the same as in the previous experiment. The enlarged spectra of Dip 2 in Figure 7b specify the redshift trend of resonant wavelength with temperature increasing, which is coincident with the conjecture. Additionally, the loss intensity retains marginal fluctuation with temperature change, as shown in the inset of Figure 7b. Compared to the amplitude variations of resonant dips caused by curvature change, that induced by temperature is negligible, which means that the proposed HCBF-based sensor can realize temperature-insensitive curvature sensing through intensity demodulation.

**Figure 8:**
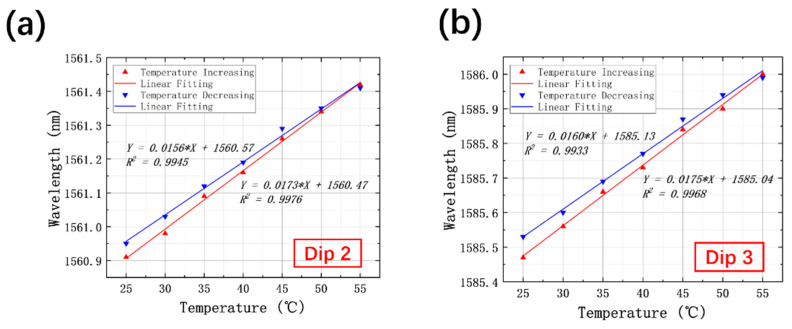
Relationship between the wavelength of resonant dips and temperature with temperature increasing and decreasing for (**a**) Dip 2 and (**b**) Dip 3.

The linear response of resonant wavelengths of Dip 2 and Dip 3 with the change of temperature was also collected. Figure 8a reveals that there has been a gradual increase in resonant wavelength of Dip 2 when the temperature rises. The fitting slope is calculated to be 17.3 pm/°C with a high linearity of 0.9976. In contrast, the drop in temperature gives rise to the blueshift of the resonant wavelength, presenting the sensitivity of 15.6 pm/°C, with an adjusted R square value of 0.9945. For Dip 3, similar results have been gained, and the temperature sensitivities are measured as 17.5 pm/°C for temperature increasing and 16.0 pm/°C for temperature decreasing, with adjusted R square values of 0.9968 and 0.9933, respectively. To conclude, both resonant dips possess reasonable linearity for temperature sensing.

## 4. Demodulation for Cross-Sensitivity of Curvature and Temperature

**Figure 9:**
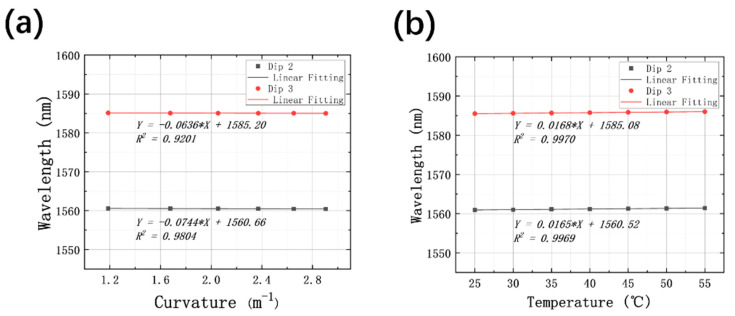
(**a**) Recalculated relationship between the wavelength of resonant dips and curvature of Dip 2 and Dip 3; (**b**) recalculated relationship between the wavelength of resonant dips and temperature of Dip 2 and Dip 3.

To monitor the curvature and temperature simultaneously, sensitivities and linearities of the HCBF-based sensor are recounted for responses of curvature and temperature by taking the average of two fitting lines collected via the measurands increasing and decreasing, as plotted in Figure 9a,b. It can be seen that recalculated curvature sensitivities are −74.4 pm/m^−1^ and −63.6 pm/m^−1^, with linearities of 0.9804 and 0.9201 for Dip 2 and Dip 3, respectively. As for temperature sensing, the average values of the slopes are 16.5 pm/°C and 16.8 pm/°C for Dip 2 and Dip 3, respectively, with linearities of 0.9969 and 0.9970.

Then, the simultaneous curvature and temperature measurement can be realized using the 2×2 matrix, which depends on the linear relationship between the resonant wavelength shift and alterations of curvature and temperature. The demodulation matrix can be defined as follows:

(4) $$\left[\begin{array}{c} \Delta \lambda_{2}\\ \Delta \lambda_{3} \end{array}\right]=\left[\begin{array}{cc} \varepsilon_{T2} & \varepsilon_{c2}\\ \varepsilon_{T3} & \varepsilon_{C3} \end{array}\right]\left[\begin{array}{c} \Delta T\\ \Delta C \end{array}\right]=\left[\begin{array}{cc} 16.5\;\mathrm{pm}/{}^{\circ}C & -74.4\;\mathrm{pm}/m^{-1}\\ 16.8\;\;\mathrm{pm}/{}^{\circ}C & -63.6\;\mathrm{pm}/m^{-1} \end{array}\right]\left[\begin{array}{c} \Delta T\\ \Delta C \end{array}\right],$$

where $\Delta \lambda_{2}$ and $\Delta \lambda_{3}$ represent wavelength shifts of Dip 2 and Dip 3, $\varepsilon_{T2}$, $\varepsilon_{T3}$ $\varepsilon_{c2}$, and $\varepsilon_{C3}$ are sensitivities of temperature and curvature, and ΔT and ΔC denote changes in temperature and curvature, respectively. Finally, changes in temperature and curvature can be obtained by inversing the matrix in Equation (4), and the rearranged matrix can be expressed as

(5) $$\left[\begin{array}{c} \Delta T\\ \Delta C \end{array}\right]=\frac{1}{200.52}\left[\begin{array}{cc} -63.6\;{}^{\circ}C/pm & 74.4\;{}^{\circ}C/\mathrm{pm}\\ -16.8\;m^{-1}/\mathrm{pm} & 16.5\;m^{-1}/\mathrm{pm} \end{array}\right]\left[\begin{array}{c} \Delta \lambda_{2}\\ \Delta \lambda_{3} \end{array}\right]$$

Undoubtedly, the simultaneous monitoring of temperature and curvature by the four-bilayer HCBF-based sensor can be effectively realized with the simple demodulation method. However, the sensitivity and linearity are not ideal enough compared to other works. Hence, higher sensitivity with enhanced stability and reliability is the motivating endeavor in the future, by optimizing the length of HCBF and addressing fabrication errors.

## 5. Conclusions

In conclusion, an ARROW–HCBF-based sensor is proposed for simultaneous measurement of curvature and temperature by simply sandwiching a segment of HCBF between two SMFs. The four-bilayer Bragg structure with the weak-light-confined property can generate a transmission spectrum of high visibility. According to the resonant wavelength shift, the measured curvature sensitivity of the HCBF-based sensor is 74.4 pm/m^−1^ in the range of 1.1859–2.9047 m^−1^, with a linearity of 0.9804, and the sensitivity of temperature varying from 25 to 55 °C is 16.8 pm/°C, with a linearity of 0.997. Then, the 2×2 matrix was adopted to demodulate the cross-sensitivity of temperature and curvature using different sensitivities of resonant dips. In addition, the HCBF-based sensor can be developed as a temperature-insensitive curvature sensor by means of resonant dip-intensity demodulation. Moreover, the proposed sensor is potentially applied for in situ structure–health monitoring and harsh-environmental sensing profiting from low transmission loss with long propagation distance and durability. The breakthrough advances, advantages of novel configuration, compact size, cost-effective production, and simplicity will make the ARROW–HCBF-based sensor continuously progress in the sensing area.
